# Effects of Bio-Banding upon Physical and Technical Performance during Soccer Competition: A Preliminary Analysis

**DOI:** 10.3390/sports7080193

**Published:** 2019-08-14

**Authors:** Will Abbott, Stuart Williams, Gary Brickley, Nicholas J Smeeton

**Affiliations:** 1 Brighton and Hove Albion Football Club, Brighton BN15 9FP, UK; 2 Centre for Sport and Exercise Science and Medicine, University of Brighton, Eastbourne BN20 7UR, UK

**Keywords:** maturation, bio-banded, chronological, match, physical development

## Abstract

Bio-banded competition has been introduced to address the variation in physical maturity within soccer. To date, no research has investigated the effect of bio-banded competition relative to chronological competition. The current study investigated the effect of bio-banding upon physical and technical performance in elite youth soccer athletes. Twenty-five male soccer athletes (11–15 years) from an English Premier League soccer academy participated in bio-banded and chronological competition, with physical and technical performance data collected for each athlete. Athletes were between 85–90% of predicted adult stature, and sub-divided into early, on-time and late developers. For early developers, significantly more short passes, significantly less dribbles and a higher rating of perceived exertion (RPE) were evident during bio-banded competition compared to chronological competition (*p* < 0.05). Significantly more short passes and dribbles, and significantly fewer long passes were seen for on-time developers during bio-banded competition (*p* < 0.05). For late developers, significantly more tackles, and significantly fewer long passes were evident during bio-banded competition (*p* < 0.05). No significant differences in physical performance were identified between competition formats. Results demonstrated that bio-banded competition changed the technical demand placed upon athletes compared to chronological competition, without reducing the physical demands. Bio-banded competition can be prescribed to athletes of differing maturation groups dependent upon their specific developmental needs.

## 1. Introduction

The large variation in physical maturity between athletes undergoing puberty creates challenges within the talent identification process. Soccer clubs potentially overlook talented youth athletes because, in competition, the physically mature may stop technical skills emerging in the physically less mature [1]. To allow equal opportunity and reduce injury risk, many sports have grouped athletes by age and body mass [2,3]. Body mass has a negligible impact upon soccer performance, however, and grouping athletes in this manner would have limited practical value due to large variations in positional requirements [4,5]. An alternative solution involves bio-banding athletes into maturation groups, or grouping based upon biological age [6,7]. Bio-banding is thought to reduce the variance in physical attributes between teams, resulting in competitive equity [7]. Controlling for maturation has led to suggestions of reduced injury risk; however, this is yet to be established. An aim of bio-banding is to support late-developing athletes who are denied opportunities to compete in competition due to the greater physical maturity of their peers. The desired outcome is a reduction of selection bias towards early developing athletes, who may not be as competent when physical attributes are controlled for [7].

From a theoretical perspective, the positive effects of bio-banding can be understood using a constraints-based framework [8]. The framework states an athlete’s motor performance results from the interactions of task, individual and environmental constraints. Task constraints relate to the specific performance context and may be the rules specifying behavior, the task goal, or task-related implements. Individual constraints consist of the individual’s characteristics, all of which can influence performance at any given moment. Examples include developmental and maturational factors. Finally, environmental constraints specify the general factors influencing motor performance, including weather conditions, as well as socio-culture and economic factors. From the interaction of these constraints, the emergent behavior is seen. Certain types of motor behavior may only emerge when particular constraints limiting performance are removed. Here, we argue that soccer athletes playing in chronological competition may lead to the motor performance of late developers being constrained by the presence of early developers. Consequently, if there is a change in the task constraint of athlete maturation as seen in bio-banded competition, then a different motor performance may emerge.

Despite the strong rationale for bio-banding, there has been no investigation into the effects of bio-banding on physical and technical performance within sport. Buchheit and Mendez-Villaneuva [9] investigated the effects of age, maturity, and body dimensions on competitive running performance in U15 soccer athletes. They concluded older, more mature athletes consistently outperformed younger, less mature teammates during chronological competition. This complimented previous research suggesting age and maturation positively impact running performance [10]. With maturational differences in physical outputs evident within chronological competition, rationale exists for investigation into the physical effects of bio-banding competition. When considering technical demands during soccer competition, Cumming et al. [11] recently investigated Premier League academy players’ experiences of participating in bio-banded competition. Results identified that early developers cited bio-banded competition as a superior physical challenge and learning stimulus compared to their chronological competition. Early developers felt there was an increased emphasis upon technique, tactics, and teamwork. Essentially, bio-banded competition exposed early developers to the challenges typically encountered by late developers. Late developers described bio-banded competition as less physically challenging compared to chronological competition. Late developers stated bio-banded competition provided them with a greater opportunity to utilise their technical, physical, and psychological attributes, and exert their influence upon competition. These findings can be interpreted using the constraints-based model of learning [8], whereby the constraint of maturation has an effect upon the emergent behavior; in this case, the style of play and tactics adopted. Cumming et al. [11] was the first to provide feedback on the effect of bio-banded competition; however, only focused upon the qualitative views of athletes. Research is yet to quantitatively investigate the effects of bio-banding upon physical and technical performance. 

The current aim was to determine differences in physical and technical performance during bio-banded and chronological soccer competition. Physical and technical performance was assessed between competition format for early-, on-time-, and late-developing athletes. It was predicted that bio-banded competition would increase the physical demands elicited upon early developers, and reduce the demands elicited upon late developers when compared to chronological competition. This effect would be reflected by higher rating of perceived exertion (RPE) values reported for early developers and the effect reversed for the late developers. Furthermore, it was predicted that bio-banding would change the maturation constraint on technical performance, and as a result, change the frequency that technical performance indicators were observed. 

## 2. Materials and Methods

### 2.1. Experimental Approach to the Problem

Physical and technical performance data was collected for 25 male soccer athletes. Athletes were aged 11–15 years, and 85–90% of predicted adult stature. Athletes were sub-divided into three maturation groups (early, on-time, and late developers) using Maturity Z-scores, and completed one bio-banded competition format, and one chronological age group competition format. Four physical performance metrics, and six technical performance metrics were analysed to determine differences between maturation group, competition format, and the interaction. Physical data was collected utilising a 10 Hz global positioning system (GPS) and 100 Hz accelerometer devices (OptimEye S5B, Version 7.18; Catapult Innovations, Melbourne, Australia). Technical data was collected using video recordings (Sony HDR CX570, Sony, Tokyo, Japan) and coding (Version 10.3.36, Sportscode Elite Software). 

### 2.2. Participants

Twenty-five male soccer athletes (age 12.7 ± 1.0 years, standing height 155.9 ± 2.9 cm, body mass 44.6 ± 5.5 kg, percentage predicted adult height achieved 86.6% ± 1.3%) were recruited from a Premier League Category One soccer academy to play in bio-banded, and chronological competition. Participants were grouped chronologically into under-12 (n = 8), under-13 (n = 9), under-14 (n = 4), and under-15 (n = 4) age groups. The relevant chronological age groups were formed in conjunction with the English Football Association rules for youth competition, and determined by the participant’s age on midnight of 31 August. Participants were also sub-divided into three maturation groups, early developers (Maturity Z-score > 1.0), on-time developers, (Maturity Z-score −1.0 to +1.0), and late developers (Maturity Z-score < 1.0). Maturity Z-scores were calculated using participant’s percentage predicted adult height, and age- and sex-specific reference values as previously utilised by Cumming et al. [11,12,13]. Participant characteristics within the maturation groups are presented in Table 1. 

Data collection occurred during the competitive season, with participants having trained 2–3 times and participated in competition once per week for a minimum of one season. All participants had been members of the academy for at least one year prior to the study and had experience of competitive academy soccer. Prior to the commencement of the study, participants and parents/guardians were provided details on the nature of the study. As participants were under the age of 18, parental or guardian consent was collected, with assent obtained by the participants. The study was conducted with the protocol being fully approved by the ethical review board at the University of Brighton prior to commencing. The study conformed to the requirements stipulated by the Declaration of Helsinki, and all health and safety procedures were complied with during the study.

### 2.3. Procedures

The Khamis–Roche equation was used to predict adult height and calculate the subsequent percentage of predicted adult height for each participant. Following calculation, current standing height has typically been reported as a percentage of predicted adult height to provide an estimation of maturation status [14]. This equation utilises current chronological age, standing height, body mass, and mid-parent standing height, of which information was measured and recorded for each individual. Trained academy staff measured participant’s standing height and body mass within two weeks of competition. Staff were ISAK Level 1 accredited and used standardised ISAK measurement techniques. Standing height was measured using a stadiometer (217 Stable Stadiometer, Seca, Hamburg, Germany). Body mass was measured using scales (875 Flat Scales, Seca, Hamburg, Germany). Parental heights were self-reported via survey and adjusted for over estimation as previously cited by Cumming et al. [11]. Adjustments were based upon measurements and self-reported heights of adults in the USA [15]. The error between predicted and actual standing height at 18 years of age is reported to be 2.1% [16]. 

Using calculations of percentage of predicted adult height, each participant was bio-banded into maturation groups. The current study focused upon the 85–90% predicted adult height maturation band. Rationale for the 85–90% maturation band is the representation of both late childhood and the onset of the pubertal growth spurt, typically occurring at approximately 86% of predicted adult height [11]. Participants competed in bio-banded competition against external opposition (aged 11–15 years) grouped by the same maturation band (85–90% predicted adult height). Bio-banded competition was played in 11 vs. 11 format, with four 20-min quarters. Matches were played on full-sized standard grass pitches (100 × 64 m), with full-sized goals (7.32 × 2.44 m). Prior to competition, participants completed a standardised warm up including physical, technical, and tactical preparation. Following bio-banded competition, participants reverted back to their chronological groups. Within two weeks of bio-banded competition, athletes participated in chronological competition against athletes of the same chronological age (under-12, under-13, under-14, and under-15 age groups). Playing formats for chronological and bio-banded competition were identical, although participants were more familiar with their teammates during chronological competition having participated in this format on a weekly basis. Physical and technical data was recorded for each participant for both bio-banded and chronological competition. 

### 2.4. Physical Data Analysis

Physical performance data was collected using a portable 10 Hz GPS and 100 Hz accelerometer devices (OptimEye S5B, Version 7.18; Catapult Innovations, Melbourne, Australia). 10 Hz GPS devices have the ability to repeatedly report short distances at high speeds with good to moderate intra-unit reliability (coefficient of variation, 5.1%), whilst demonstrating coefficient of variations of 1.2–6.5% for acceleration and deceleration [17,18]. GPS devices were worn in a designated tight-fitting vest located between the scapulae. GPS devices were switched on 15 min prior to the warm up, in accordance with manufacturer’s instructions, and switched off immediately following competition. Participants wore the same GPS device for competition, avoiding inter-device error. Following data collection, GPS data was downloaded to a PC and analysed using Catapult Sprint software (Catapult Sprint 5.1.5, Catapult Innovations, Melbourne, Australia). Once downloaded, competition data was edited and split into four 20-min quarters. Only participants completing the entire match were included within the analysis process. The mean number of satellites, and the horizontal dilution of position were recorded during data collection. If values ranged <12 for number of satellites, or >1 for horizontal dilution of position, data was excluded. Sessional RPE was recorded using the modified Borg CR10-scale. RPE values were recorded 30-min following the cessation of competition. Participants were familiar with the RPE scale, having been exposed to the scale for at least a year prior to the data collection period. Descriptions of physical performance metrics recorded during the study are shown in Table 2.

### 2.5. Technical Data Analysis

Both bio-banded and chronological competition formats were recorded using a camcorder (Sony HDR CX570). Recordings were coded and analysed by two trained academy performance analysts (mean experience 8.5 ± 2.1 years) using SportsCode (Version 10.3.36, Sportscode Elite Software, Sportstec Ltd, Geluksburg, South Africa). The technical performance metrics coded and analysed are shown in Table 2. Definitions of technical performance metrics were determined using existing criteria at the professional football club. 

### 2.6. Statistical Analysis

Data was analysed for normal distribution using Kolmogorov–Smirnov and Shapiro–Wilk tests. To investigate differences in physical and technical performance between bio-banded and chronological competition for early, on-time, and late developers, two-way mixed design ANOVAs were used where Competition Format (Bio-banded, Chronological) was the within-subjects variable, and Maturation Group (Early, On-Time, Late) was the between-subjects variable. Eta-squared values were calculated to estimate the effect size for the ANOVA. An eta-squared effect size of *η*^2^ = 0.01 was considered a small effect size, an effect size of *η*^2^ = 0.09 was considered a medium effect size, whilst *η*^2^ = 0.25 was considered a large effect size. Bonferroni tests were used post-hoc to assess where differences occurred, with Cohen’s *d* tests were used to calculate effect sizes. An effect size of *d* = 0.2 was considered a small effect size, an effect size of *d* = 0.5 was considered a medium effect size, whilst *d* = 0.8 was considered a large effect size. All statistical analyses were performed using the software IBM SPSS statistics (version 22; SPSS, IBM, Chicago, IL, USA). The level of statistical significance was set at *p* < 0.05. 

## 3. Results

For physical and technical metrics, analysis was initially performed with age group as an independent variable. However, no significant effects or interactions were identified for age group, and consequently, age group was removed as a factor.

### 3.1. Physical Analysis

Mean RPE produced by different maturation groups during competition formats are presented in Table 3. Significant differences were identified between maturation group (F_(2,22)_ = 9.56; *p* < 0.05, *η*^2^ = 0.47). Follow up analysis demonstrated late developers produced significantly higher RPE when compared to early developers (*p* < 0.05). Significant differences were also identified in the interaction between competition format and maturation group (F_(2,22)_ = 17.49; *p* < 0.01, *η*^2^ = 0.61). Results demonstrated that early developers produced significantly higher RPE in bio-banded competition compared to chronological (t = 9.30; *p* < 0.05, *d* = 1.2). There were no significant differences between RPE produced by on-time developers, or late developers within competition formats. 

Alongside RPE, Table 3 presents mean total, high-speed running, and explosive distances produced during competition formats for different maturation group. For total distance, no significant differences were identified between competition format, or the interaction between competition format and maturation group. Significant differences were identified between maturation group (F_(2,22)_ = 43.92; *p* < 0.01, *η*^2^ = 0.80) however. Follow-up analysis demonstrated late developers produced significantly higher total distances compared to early developers (*p* < 0.01). There were no significant differences between late and on-time developers, or on-time and early developers. For high-speed running distance, no significant differences were identified between competition format, maturation group, or the interaction. For explosive distance, no significant differences were identified between competition format, or the interaction between competition format and maturation group. Significant differences were identified between maturation group (F_(2,22)_ = 12.48; *p* < 0.01, *η*^2^ = 0.53), with late developers producing significantly higher explosive distances compared to early and on-time developers (*p* < 0.05). 

### 3.2. Technical Analysis

**Figure 1:**
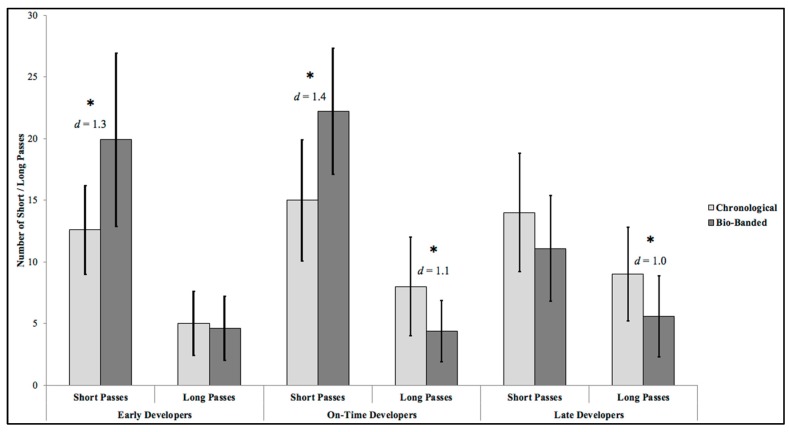
Mean number of short and long passes produced by early-, on-time-, and late-developing athletes during bio-banded and chronological competition formats. N.B. asterisk represents significant difference between competition formats (*p* < 0.05).

Mean short passes produced by maturation groups during competition formats are presented in Figure 1. Significant differences were identified between competition formats (F_(1,22)_ = 16.06; *p* < 0.05, *η*^2^ = 0.42), and the interaction between competition format and maturation group (F_(2,22)_ = 4.18; *p* < 0.05, *η*^2^ = 0.53). Follow-up analysis demonstrated the bio-banded format produced significantly more short passes than chronological, specifically for early (t = 4.99; *p* < 0.05, *d* = 1.3) and on-time developers (t = 5.13; *p* < 0.05, *d* = 1.4).

Figure 1 also demonstrates the mean long passes produced during competition formats for the maturation groups. As with short passes, significant differences were identified between competition formats (F_(1,22)_ = 19.28; *p* < 0.01, *η*^2^ = 0.47), and the interaction between competition format and maturation group was significant (F_(2,22)_ = 3.09; *p* < 0.01, *η*^2^ = 0.22). Follow-up analysis demonstrated the chronological format produced significantly more long passes than bio-banded, for on-time (t = 3.55; *p* < 0.01, *d* = 1.1) and late developers (t = 3.32; *p* < 0.05, *d* = 1.0) only.

Mean dribbles produced by maturation groups during competition formats are presented in Table 4. There was a significant interaction between competition format and maturation group (F_(2,22)_ = 8.69; *p* < 0.01, *η*^2^ = 0.44). Follow-up analysis demonstrated the bio-banded format produced significantly more dribbles than the chronological format for on-time developers (t = 7.24; *p* < 0.01, *d* = 0.7), but significantly less dribbles for early developers (t = 2.52; *p* < 0.05, *d* = 0.8). 

Table 4 presents mean number of tackles produced during competition formats for maturation groups. Significant differences were identified for the interaction between competition format and maturation group (F_(2,22)_ = 6.20; *p* < 0.01, *η*^2^ = 0.36). Follow-up analysis demonstrated the bio-banded format produced significantly more tackles than the chronological format for late developers (t = 3.57; *p* < 0.01, *d* = 1.0). No significant differences were identified between competition formats for early or on-time developers. 

Alongside dribbles and tackles, Table 4 also presents mean number of shots and crosses produced during competition formats for maturation groups. For both technical metrics, no significant effects were identified between competition format, maturation group, or the interaction.

## 4. Discussion

The current study was the first to objectively assess physical and technical performance between bio-banded and chronological competition. In support of the predicted effects of bio-banded competition on physical performance, early developers produced significantly higher RPE during bio-banded competition when compared to chronological. However, no significant differences were identified in RPE produced between competition formats, for on-time or late developers. Additionally, no significant differences were identified in total, high-speed running, or explosive distances produced between competition formats for any maturation group. In support of the technical performance predictions, significant differences were identified between competition formats. There was an increased frequency of short passes in early and on-time developers, and a decrease in long passes for on-time and late developers, during bio-banded competition. Dribbles decreased in early developers and increased in on-time developers. There were increased tackles performed by late developers in bio-banded competition compared to chronological. 

Results demonstrated that late developers produced significantly higher RPE when compared to early developers overall. The higher RPE produced by late developers is likely the result of a habitual exertion response to competing with and against athletes of a higher relative physical development during usual chronological competition. When investigating differences in RPE between competition formats, results demonstrated early developers produced significantly higher RPE during bio-banded competition compared to chronological. No differences were identified between competition formats for other maturation groups. Higher RPE during bio-banded competition is likely the result of the increased perceived physical demands when competing against other early developers. Current results compliment research conducted by Cumming et al. [11], finding early developers described bio-banded competition as a superior physical challenge compared to chronological competition. Additionally, late developers described bio-banded competition as less physically challenging in comparison to chronological competition. Significant differences in physical performance of maturation groups were identified in the current study. Late developers produced significantly higher total and explosive distance when compared to early developers overall. From a constraints-based framework [8], the learnt response towards higher total and explosive distances found in late developers would emerge from previous exposure to the task constraints imposed by the early developers during competition. For late developers to maintain their competitiveness, they would be required to produce higher total and explosive distances, whereas the early developers would not need to do so. When considering differences in physical performance between competition formats, no significant differences were identified for total, high-speed running or explosive distances for any maturation group. 

When investigating short and long passes produced between competition formats, significant differences were identified. Bio-banded competition produced significantly higher number of short passes for early and on-time developers, when compared to chronological. For long passes, bio-banded competition produced significantly less for on-time and late developers, when compared to chronological competition. For number of dribbles, bio-banded competition produced significantly more dribbles compared to chronological for on-time developers, but less dribbles for early developers. For number of tackles, bio-banded produced significantly more tackles for late developers only. Referring to the constraints-based model, bio-banded competition resulted in more tackles and fewer long passes performed by late developers because of the reduced physical maturation of opponents compared to usual chronological competition. Presumably, decreased relative strength of opponents enable tackling behavior to emerge. Likewise, the advantage of a long pass to a more physically mature teammate to ‘hold the ball up’ is no longer an option in this format. Consequently, this long pass tactical option is no longer exploited. In the case of early developers, the opportunity to dribble around less physically mature opponents is reduced during bio-banded competition, and therefore, this behavior was less frequently observed. The necessity to produce more short passes to move the ball towards the opponent’s goal is required, and emerges in this competition format. An alternative explanation to the aforementioned result of the increased frequency of short passes is the result of a lack of team familiarity during bio-banded competition. With athletes are opting for shorter, simpler passes to maintain possession. Increases in dribble frequency during bio-banded competition for on-time developers could represent a lack of understanding with teammates, resulting in athletes being isolated with possession of the ball.

The finding of bio-banding altering the frequency of technical actions demonstrates a potential method of manipulating technical performance during youth soccer competition. Rampanini et al. [21] identified successful short passes, dribbles and tackles as important technical skill parameters for success in top-level professional soccer. Additionally, Bradley et al. [22] identified a superior technical proficiency in the Premier League when comparing to the lower English leagues. This was characterised by significantly higher numbers of total passes in the Premier League, in comparison to a more transient long-ball tactic utilised at lower standards of soccer [23]. Current results compliment research by Cumming et al. [11], and suggest bio-banded competition elicits a distinct technical stimulus for early-, on-time-, and late-developing athletes relative to chronological competition. 

Differences between bio-banded and chronological competition formats were not observed when investigating the number of shots and crosses performed. For the number of shots, there were no significant differences between competition formats for any maturation group. This finding was repeated for the number of crosses. The lack of significant differences identified between competition formats for these two technical metrics is likely the result of the specificity of actions to playing positions. Shooting is an action more commonly associated with strikers and attacking playing positions, and infrequently seen in defenders. Crossing is a technical action frequently exhibited in wide attacking and wide defending playing positions, but rarely seen for central defenders. Due to the limited sample size, analysis was not conducted for individual playing position, and therefore, the large variations between playing positions may have resulted in no significant differences being identified. This is a topic requiring further investigation. 

The current study provides applied practitioners with valuable insights regarding the physical and technical differences between competition formats for differing maturation groups. Despite the aforementioned advantages, bio-banded competition is not recommended as a replacement for chronological competition. To ensure holistic development of youth athletes and encourage observation from coaches and scouts in varied environments, it is recommended bio-banded competition be used as an adjunct to chronological competition. Bio-banding is suggested to be of most value at the onset of adolescence, when maturity selection biases begin to emerge. Additional to the physical and technical demands discussed within the current study, Reeves et al. [1] state it is vital to consider the psychological and social impact of bio-banding. Cumming et al. [5] found late developers possessed a psychological advantage characterised by greater self-regulation, the result of regularly competing against relatively older athletes in chronological competition. Inclusion of bio-banded and chronological competition within the development programme provides continued exposure to a variety of stimuli important for the holistic development of youth athletes.

It is important to consider the limitations of this preliminary study. Firstly, the study was conducted using youth soccer athletes at a Premier League academy and may not be directly applicable to other clubs or levels. Secondly, due to the nature of the study, a small sample size was recruited, and each athlete only completed one bio-banded and one chronological competition. This prevented further investigation into the effect of bio-banded and chronological competition formats for different playing positions and maturation groups. The current study found no significant differences in shooting and crossing metrics between competition formats. Considering the specificity of these two technical metrics to playing positions, conducting positional analysis may have resulted in further findings. Finally, it is important to acknowledge that physical and technical performances vary dependent upon opposition, tactics, score line, and other contextual factors [24,25]. Considering athletes only completed one of each competition formats, and given the high match-to-match variability of physical and technical performance [26], caution must be adopted when interpreting the current results. 

## 5. Conclusions 

The current study has significant applications for practitioners working within youth soccer. Results provide support for bio-banding as an intervention to alter the task constraints of youth soccer competition. It is concluded that bio-banded competition places a unique technical demand upon athletes of differing maturation groups when compared to chronological formats, without reducing the physical demands. Results can aid applied practitioners in individualising the prescription of competition formats to different maturation groups, dependent upon their physical and technical development needs. Inclusion of bio-banded competition as an adjunct to the existing programme would allow youth athletes of varying maturation groups to develop a variety of technical skills, encouraging their holistic development. 

## Figures and Tables

**Table 1:** Participant characteristics of maturation groups (mean ± SD).

Maturation Group.	Number of Participants (n)	Age (Years)	Maturity Z-score	Percentage Predicted Adult Height (%)	Height (cm)	Weight (kg)	Playing Position (n)
Early Developers	8	11.5 ± 0.2	1.5 ± 0.4	87.2 ± 1.2	158.2 ± 5.7	44.3 ± 5.2	DEF = 2 MID = 3 FOR = 3
On-Time Developers	10	12.3 ± 0.3	−0.2 ± 0.7	86.2 ± 0.9	156.8 ± 4.3	41.8 ± 3.9	DEF = 4 MID = 4 FOR = 2
Late Developers	7	13.8 ± 0.6	−1.7 ± 0.4	86.8 ± 1.7	155.7 ± 4.6	46.9 ± 5.5	DEF = 2 MID = 3 FOR = 2

DEF = Defenders, MID = Midfielders, FOR = Forwards.

**Table 2:** Descriptions of physical and technical performance metrics collected during competition formats.

Performance Metrics	Description
**Physical Performance Metrics**	
Total Distance (m)	The total distance travelled.
High-Speed Running Distance (m)	The distance travelled >5.5 m.s^−1^ [19].
Explosive Distance (m)	The distance travelled accelerating >2 m.s^−2^, and the distance travelled decelerating <−2 m.s^−2^ added together [20].
Rating of Perceived Exertion (RPE)	Subjective rating of exertion using the modified Borg CR1-10-scale
**Technical Performance Metrics**	
Shot	A successful strike of the ball aimed at the opposing goal.
Short pass	A strike of the ball (<20 m in distance) directed at a teammate, and that was successfully controlled.
Long pass	A strike of the ball (>20 m in distance) directed at a teammate, and that was successfully controlled.
Cross	A successful long pass from the widest quarter of the pitch landing in the opposition penalty area.
Dribble	Successfully running past an opponent with the ball.
Tackle	A successful attempt to remove the ball from the opponent’s possession through a physical challenge.

**Table 3:** Mean (± SD) physical performance metrics produced by early, on-time, late developing athletes during bio-banded and chronological competition formats. N.B. asterisk represents significant difference (*p* < 0.05).

Physical Performance Metric.	Early Developers			On-Time Developers			Late Developers		
	Chronological	Bio-Banded	All Formats	Chronological	Bio-Banded	All Formats	Chronological	Bio-Banded	All Formats
Total Distance (m)	7942.9 ± 369.1	8254.6 ± 272.3	8098.7 ± 351.1	8583.1 ± 337.7	8656.8 ± 281.3	8620.0 ± 304.9	9083.8 ± 248.9	8971.9 ± 329.5	9027.8 ± 287.9
High-Speed Running Distance (m)	757.1 ± 94.2	783.4 ± 75.7	770.3 ± 83.2	773.8 ± 104.9	755.5 ± 78.8	764.7 ± 90.8	848.3 ± 92.7	813.5 ± 81.2	830.9 ± 86.5
Explosive Distance (m)	422.0 ± 36.8	455.1 ± 26.0	438.6 ± 35.1	466.7 ± 24.5	476.0 ± 31.8	471.4 ± 28.1	535.9 ± 70.5	486.0 ± 40.3	530.8 ± 52.2
RPE	6.6 ± 0.5	7.5 ± 0.9	7.4 ± 1.1	8.3 ± 0.7	7.5 ± 0.9	7.9 ± 0.9	9.0 ± 0.5	8.4 ± 0.9	8.7 ± 0.8

**Table 4:** Mean (± SD) number of dribbles, tackles, shots and crosses produced by early-, on-time-, and late-developing athletes during bio-banded and chronological competition formats. N.B. asterisk represents significant difference (*p* < 0.05).

Technical Performance Metric	Early Developers		On-Time Developers		Late Developers	
	Chronological	Bio-Banded	Chronological	Bio-Banded	Chronological	Bio-Banded
Dribbles	7.7 ± 2.1	6.0 ± 2.2	3.0 ± 2.3	4.6 ± 2.5	3.0 ± 1.6	4.9 ± 3.2
Tackles	9.0 ± 3.7	9.0 ± 3.3	7.6 ± 3.0	8.0 ± 3.8	4.4 ± 2.7	7.5 ± 3.4
Shots	1.9 ± 1.5	2.7 ± 1.6	2.5 ± 1.8	2.5 ± 2.0	2.4 ± 1.7	2.5 ± 1.6
Crosses	1.9 ± 1.9	2.0 ± 1.7	1.9 ± 2.1	1.6 ± 1.6	2.0 ± 1.9	0.8 ± 0.9
